# Co-composted Biochar Enhances Growth, Physiological, and Phytostabilization Efficiency of *Brassica napus* and Reduces Associated Health Risks Under Chromium Stress

**DOI:** 10.3389/fpls.2021.775785

**Published:** 2021-11-18

**Authors:** Muhammad Naveed, Bisma Tanvir, Wang Xiukang, Martin Brtnicky, Allah Ditta, Jiri Kucerik, Zinayyera Subhani, Muhammad Zubair Nazir, Maja Radziemska, Qudsia Saeed, Adnan Mustafa

**Affiliations:** ^1^Institute of Soil and Environmental Sciences, University of Agriculture, Faisalabad, Pakistan; ^2^College of Life Sciences, Yan’an University, Yan’an, China; ^3^Department of Agrochemistry, Soil Science, Microbiology and Plant Nutrition, Faculty of AgriSciences, Mendel University in Brno, Brno, Czechia; ^4^Faculty of Chemistry, Institute of Chemistry and Technology of Environmental Protection, Brno University of Technology, Brno, Czechia; ^5^Department of Environmental Sciences, Shaheed Benazir Bhutto University, Sheringal, Upper Dir, Pakistan; ^6^School of Biological Sciences, The University of Western Australia, Perth, WA, Australia; ^7^Faculty of Life Sciences, University of Central Punjab, Lahore, Pakistan; ^8^Institute of Environmental Engineering, Warsaw University of Life Sciences, Warsaw, Poland; ^9^Biology Centre, The Soil and Water Research Infrastructure (SoWa RI), Czech Academy of Sciences, Ceske Budejovice, Czechia

**Keywords:** chromium, *Brassica*, health risks, phytostabilization, heavy metals

## Abstract

Among heavy metals, chromium (Cr) contamination is increasing gradually due to the use of untreated industrial effluents for irrigation purposes, thereby posing a severe threat to crop production. This study aimed to evaluate the potential of compost, biochar (BC), and co-composted BC on the growth, physiological, biochemical attributes, and health risks associated with the consumption of *Brassica* grown on Cr-contaminated soil. Results revealed that Cr stress (Cr-25) significantly reduced the growth and physiological attributes and increased antioxidant enzyme activities in *Brassica*, but the applied amendments considerably retrieved the negative effects of Cr toxicity through improving the growth and physiology of plants. The maximum increase in plant height (75.3%), root length (151.0%), shoot dry weight (139.4%), root dry weight (158.5%), and photosynthetic rate (151.0%) was noted with the application of co-composted BC under Cr stress (Cr-25) in comparison to the control. The application of co-composted BC significantly reduced antioxidant enzyme activities, such as APX (42.5%), GP (45.1%), CAT (45.4%), GST (47.8%), GR (47.1%), and RG (48.2%), as compared to the control under Cr stress. The same treatment reduced the accumulation of Cr in grain, shoot, and roots of *Brassica* by 4.12, 2.27, and 2.17 times and enhanced the accumulation in soil by 1.52 times as compared to the control. Moreover, the application of co-composted BC significantly enhanced phytostabilization efficiency and reduced associated health risks with the consumption of *Brassica*. It is concluded that the application of co-composted BC in Cr-contaminated soil can significantly enhance the growth, physiological, and biochemical attributes of *Brassica* by reducing its uptake in plants and enhanced phytostabilization efficiency. The tested product may also help in restoring the soils contaminated with Cr.

## Introduction

The contamination of heavy metals (HMs) in soil is a serious threat to sustainable crop production and soil quality because these metals are non-degradable and persist in soil for longer durations (Kamran et al., 2019). HMs accumulation deteriorates the physicochemical and biological properties of soil, which results in poor nutrient availability and ultimately decreased crop yield (Mehmood et al., 2018b). Naturally, HMs are the constituent of sediments, adsorbed on soil organic matter, and their toxicity increased as free ions in soil solution (Abbas et al., 2020). Modern agricultural practices, especially excessive use of inorganic fertilizers and agrochemicals, have polluted soil and ultimately resulted in enhanced environmental degradation and threatened sustainability (Ditta et al., 2015; Murtaza et al., 2021b). Moreover, the application of organic waste manure, sewage sludge, and irrigation with industrial effluents contributes significant amounts of HMs into agricultural systems (Kamran et al., 2019; Bashir et al., 2020a,b).

Among HMs, chromium (Cr) is considered a major environmental pollutant due to its severe toxicity and recalcitrant nature (Ali et al., 2018). Industrial effluents from electroplating, catalytic manufacturing and wood preservation, leather tanning, and alloy preparation contain large amounts of Cr, which are the major sources of Cr contamination in the soil (Govil and Krishna, 2018). In the environment, Cr exits in various oxidation states, but Cr(III) and Cr(VI) are dominant in soil (Junaid et al., 2016; Bashir et al., 2020a). Cr(III) is an essential micronutrient for animals and involved in cell metabolism (de Oliveira et al., 2014), relatively stable, and 10–100 times less toxic as compared to Cr(VI) (Tepanosyan et al., 2020). Cr(VI) is a highly soluble, potentially mobile across membranes with strong oxidation potential, toxic, allergenic, carcinogenic, and irritant that damages the liver, kidney, and lungs (Bashir et al., 2020a,b; Tepanosyan et al., 2020).

The application of contaminated water with a higher concentration of Cr to the agricultural land not only results in detrimental effects on plant growth and loss of production, food security, and animal health but also affects the overall ecosystem (Mehmood et al., 2021). Cr phytotoxicity severely affects seed germination, decreases root growth and shoot growth, and ultimately reduces biomass production (Bashir et al., 2020a,b). Cr disrupts photosynthesis, nutrient utilization, water relation, and enzymatic activity which produces reactive oxygen species (ROS) that oxidize lipids, protein, and nucleic acid and ultimately result in plant death (de Oliveira et al., 2014; Bashir et al., 2020b; Murtaza et al., 2021b).

Various physical (e.g., soil incineration, landfill, excavation, and soil flushing), chemical (e.g., oxidation–reduction), and biological techniques (e.g., phytoremediation and biodegradation) have been used for remediation of metal-contaminated soils or conversion into less toxic form and reduced bioavailability to plants (Mustafa et al., 2020; Sabir et al., 2020; Irshad et al., 2021). Physical and chemical remediation techniques are laborious, time-consuming, and energy-consuming, and considerable toxic waste products are the main cause of limited application despite high efficiency and utility (Mustafa et al., 2020; Sabir et al., 2020). Among organic amendments, farmyard manure, animal waste, and compost are used to reduce HMs uptake for a shorter duration because these amendments improve soil physicochemical properties, nutrient uptake, soil aggregation, and maintain soil quality (Mehmood et al., 2018a; Chukwuka et al., 2020; Hu et al., 2020; Murtaza et al., 2021a). It is, therefore, a need of the hour to develop efficient *in situ* immobilization techniques that should be cost-effective, less destructive, and environment friendly for soil restoration.

In recent years, biochar (BC) is gaining popularity for the restoration of metal-contaminated sites and carbon sequestration in the soil for longer periods due to its recalcitrant nature, cost-effective, and ecofriendly approach (Mehmood et al., 2018a; Kamran et al., 2019). BC is a fine-grained black color porous material with carbonaceous nature produced from organic wastes (e.g., crop residues, sewage slug, animal wastes, and farmyard manure) at high temperatures under limited or no oxygen supply (Abbas et al., 2020; Hu et al., 2020). BC has unique physicochemical properties such as porous structure, high surface area, variety of oxygen-containing functional groups that make it an excellent material for soil fertility improvement, carbon sequestration, wastewater treatment, metal stabilization, and organic pollutant degradation in soil (Mehmood et al., 2018a,b; Chukwuka et al., 2020; Hu et al., 2020; Murtaza et al., 2021a). BC has a stable carbon pool that influences soil physicochemical and biological properties and directly increases soil organic carbon contents (Sarfraz et al., 2019; Ijaz et al., 2020; Ullah et al., 2020). The application of BC in agricultural soil improves aeration, increases nutrient availability, and increases soil organic matter, a microbial activity that ultimately increases crop production, decreases fertilizer requirement and nutrient leaching, and reduces soil erosion by controlling soil pollution (Doumer et al., 2016; Rizwan et al., 2021). BC reduces metal mobility through physical adsorption, ion exchange, surface precipitation, and metal complexation (Sarfraz et al., 2019; Irshad et al., 2021); cation exchange; and electrostatic interaction in metal-contaminated soil (Brassard et al., 2016). The adsorption property and high carbon contents of BC make it an excellent material to minimize Cr toxicity in the terrestrial ecosystem (Jia et al., 2017).

Various studies reported that combined application of compost and BC could be a better approach as BC increases compost stability and improves the productiveness of the soil, HM adsorption, and production of crop and carbon sequestration (Agegnehu et al., 2015; Coelho et al., 2018). The mixing of BC and compost adds value in increasing soil fertility by different aspects such as reducing the volatility of ammonia from manure or minimizing methane emission (Coelho et al., 2018). Compost provides high nutrients to the soil compared to BC, whereas BC could be reducing the decomposition rate of compost (Ditta et al., 2015). Many studies showed the combined application of BC and compost had a synergistic impact in improving soil structure, water-holding capacity, and nutrient contents under field conditions with reduced fertilizer use and nutrient loss from soil (Agegnehu et al., 2015). BC co-composting can reduce compost decomposition, decrease nutrient loss in the environment, improve soil structure by direct organic matter addition, and increase microbial biodiversity and activity in the soil. We hypothesized that co-composted BC could act as a better ameliorant for improving growth, physiology, and antioxidant machinery by retrieving the harmful effects of Cr in Cr-contaminated soil. The objectives of this study were to investigate the impact of compost, BC, and co-composted BC on growth, physiological, antioxidant, and biochemical attributes of *Brassica* grown under Cr-contaminated soil and the phytostabilization efficiency of compost, BC, and co-composted BC in addition to the Cr-contaminated soil and associated health risk assessment of *Brassica* grown under Cr stress.

## Results

### Growth Attributes

**FIGURE 1:**
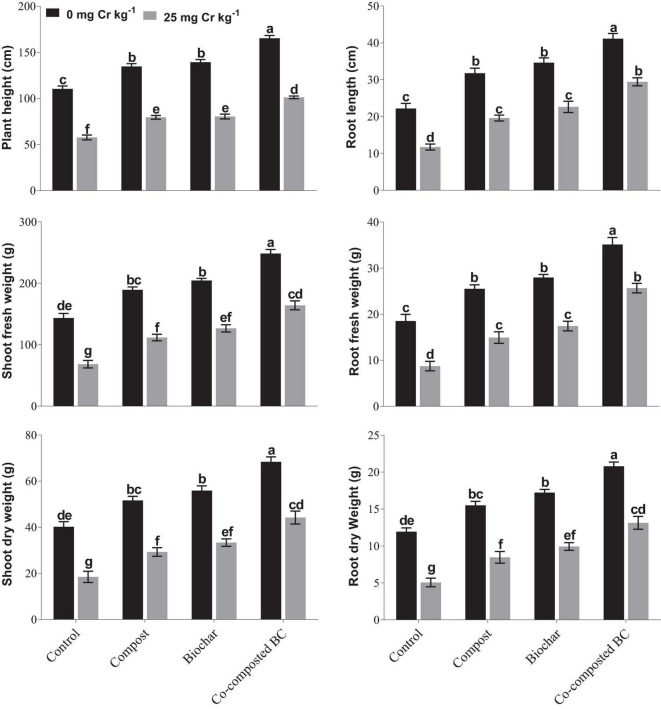
Impact of compost, biochar, and co-composted biochar on growth and yield parameters of *Brassica*. The values are presented as mean ± standard error (*n* = 3). The values sharing the same letter(s) in bars are statistically non-significant with each other at *p* < 0.05.

Results of this study showed that the application of compost, BC, and co-composted BC significantly (*p* < 0.05) improved the growth of *Brassica* under Cr contamination (Figure 1). It was observed that the application of co-composted BC (Com-BC-2) resulted in the maximum increase in plant height (49.8%), root length (85.6%), shoot fresh (72.9%) and dry weight (70.0%), and root fresh (89.6%) and dry weight (74.0%) as compared to the control under normal soil, i.e., 0 mg Cr kg^–1^ (Figure 1). Soil spiked with Cr (25 mg Cr kg^–1^) decreased the growth parameters of *Brassica*. However, the application of co-composted BC resulted in the maximum increase in shoot and root length, shoot fresh and dry weight, and root fresh and dry weight of *Brassica* by 75.3, 151.0, 140.0, 139.4, 193.9, and 158.5%, respectively, as compared to the control (Cr-25). It was followed by sole application of BC and compost in comparison to the controls under normal (Cr-0) and Cr stress (Cr-25).

### Physiological Attributes

**FIGURE 2:**
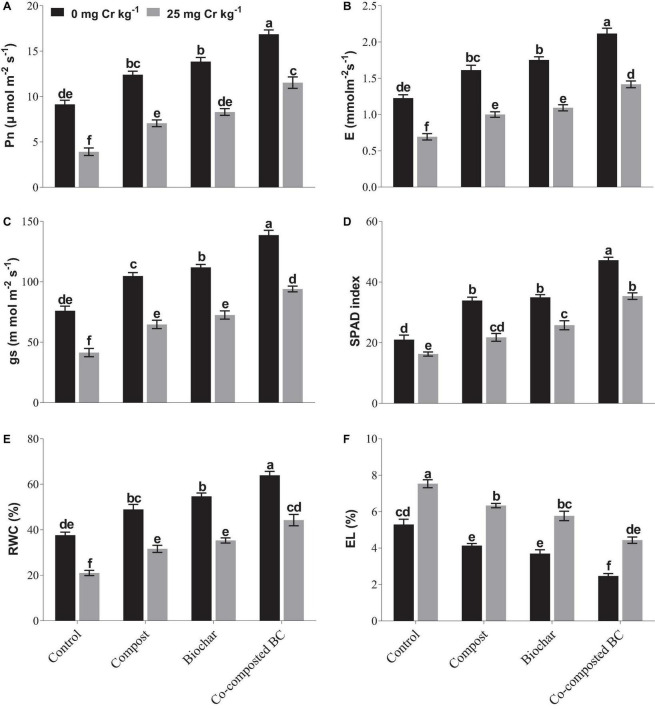
Impact of compost, biochar, and co-composted biochar on physiological parameters of *Brassica*. **(A)** Photosynthetic rate, **(B)** transpiration rate, **(C)** stomatal conductance, **(D)** chlorophyll content, **(E)** relative water content, and **(F)** electrolyte leakage. The values are presented as mean ± standard error (*n* = 3). The values sharing the same letter(s) in bars are statistically non-significant with each other at *p* < 0.05.

The application of BC and co-composted BC significantly (*p* < 0.05) enhanced the physiological attributes of *Brassica* under normal soil (Cr-0) or contaminated soil (Cr-25) when compared to the control (Figure 2). Under normal soil (Cr-0), the application of compost, BC, and co-composted BC significantly improved the physiological attributes of *Brassica* as compared to the control under Cr stress (Cr-25). In the case of chlorophyll contents (SPAD index), the maximum increase (2.25 times) was noticed with co-composted BC as compared to the control under normal soil (Cr-0). In soil spiked with Cr-25 concentration, the application of co-composted BC increased 2.17 times more chlorophyll contents (SPAD index) than the control under Cr stress (Cr-25). The same treatment caused an increase in 151.0, 104.3, 127.0, and 101.6% in photosynthetic rate, transpiration rate, stomatal conductance, and relative water contents, respectively, with the application of co-composted BC under Cr contamination (Cr-25). It was followed by BC and compost application under Cr contamination.

### Antioxidant Enzyme Activities

**FIGURE 3:**
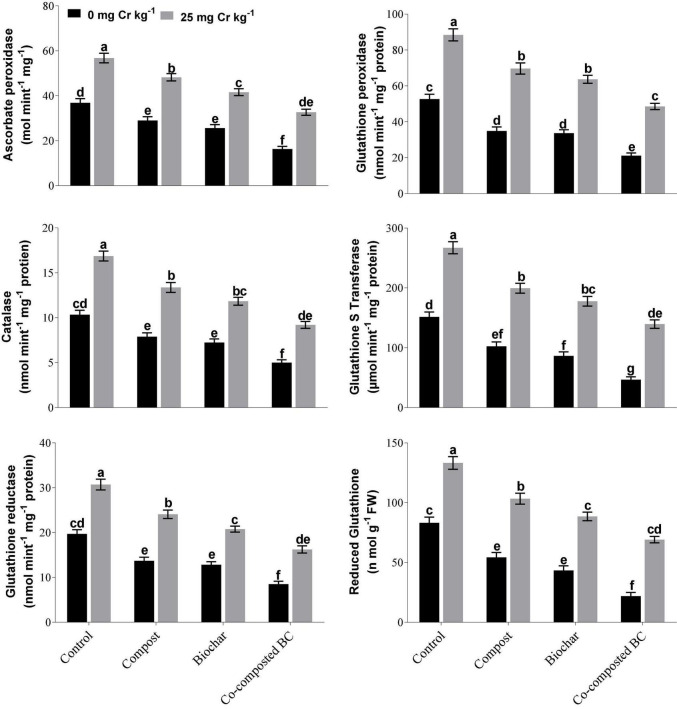
Impact of compost, biochar, and co-composted biochar on antioxidant activities of *Brassica*. The values are presented as mean ± standard error (*n* = 3). The values sharing the same letter(s) in bars are statistically non-significant with each other at *p* < 0.05.

Under Cr stress (Cr-25), the maximum APX (56.78 mol min^–1^ mg^–1^ protein), GP (88.43 mol min^–1^ mg^–1^ protein), CAT (16.87 nmol min^–1^ mg^–1^ protein), GST (267.13 μmol min^–1^ mg^–1^ protein), GR (30.70 mol min^–1^ mg^–1^ protein), and RG (133.27 nmol min^–1^ mg^–1^ protein) values were recorded without any treatment (Figure 3). The application of organic amendments (compost, BC, and co-composted BC) significantly reduced antioxidant enzyme activities as compared to the control. The maximum decrease in APX (42.5%), GP (45.1%), CAT (45.4%), GST (47.8%), GR (47.1%), and RG (48.2%) was observed with the application of co-composted BC in comparison to the control treatment.

### Bean Weight and Total Soluble Sugars

**FIGURE 4:**
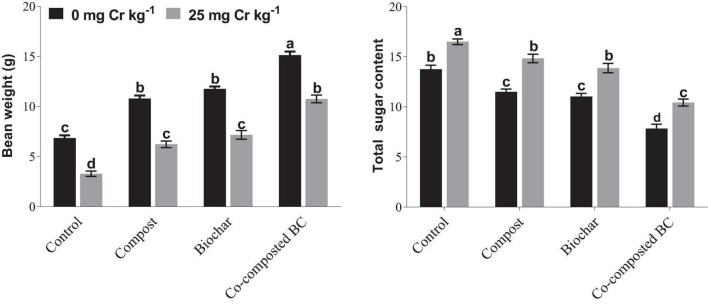
Impact of compost, biochar, and co-composted biochar on bean weight and total sugar contents (TSCs) of *Brassica napus*. The values are presented as mean ± standard error (*n* = 3). The values sharing the same letter(s) in bars are statistically non-significant with each other at *p* < 0.05.

Bean weight was significantly reduced under Cr stress, while a significant increase was noted with the application of compost, BC, and co-composted BC under Cr stress, i.e., Cr-25 (Figure 4). Under Cr stress, the minimum bean weight (3.29 g) was noted in the control treatment without any amendment, while the maximum bean weight (15.13 g) was recorded with the application of co-composted BC under normal conditions (Cr-0). The maximum bean weight (10.76) recorded with the application of co-composted BC was 3.27 times more as compared to the control (Cr-25). In the case of total sugar content (TSC), an opposite trend was recorded with the application of compost, BC, and co-composted BC (Figure 4). The maximum TSC (16.5) was observed under the control treatment (Cr-25), while the minimum TSC (7.83) was observed with the application of co-composted BC under normal conditions (Cr-0). The application of co-composted BC significantly reduced TSC (36.8%) in *Brassica* as compared to the control under Cr stress (Cr-25).

### Chromium Accumulation

**FIGURE 5:**
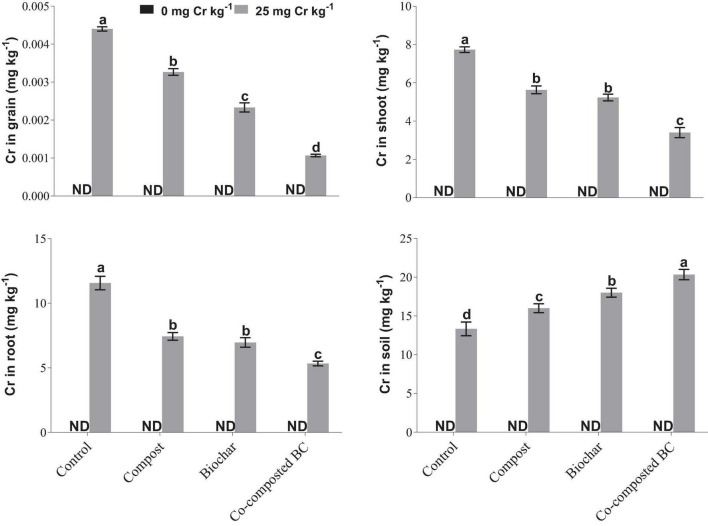
Impact of compost, biochar, and co-composted biochar on Cr accumulation in different portions of *Brassica napus*. The values are presented as mean ± standard error (*n* = 3). The values sharing the same letter(s) in bars are statistically non-significant with each other at *p* < 0.05.

**FIGURE 6:**
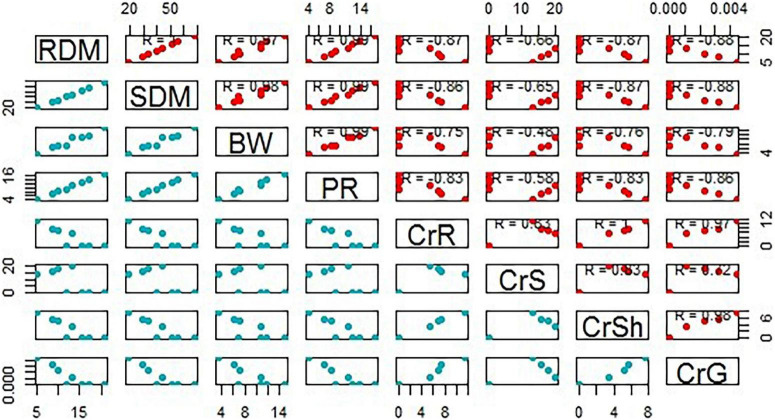
A scatter matrix plot demonstrating pairwise scatter plots of variables in a matrix format, determining variables correlation. BW, bean weight; SDM, shoot dry mass; RDM, root dry mass; PR, photosynthetic rate; CrS, Cr in soil; CrR, Cr in the root; CRSh, Cr in the shoot; CrG, Cr in grain.

With the application of compost, BC, and co-composted BC, Cr accumulation in grain, root, and shoot samples of *Brassica* was significantly reduced in comparison to the control under Cr stress (Figure 5). However, Cr accumulation in soil was significantly enhanced with the application of co-composted BC in comparison to the control under Cr stress (Cr-25). With the application of co-composted BC, the minimum accumulation in grain (1.06 μg kg^–1^ DW), shoot (3.40 mg kg^–1^ DW), and root (5.33 mg kg^–1^ DW) portions of *Brassica* were 4.12, 2.27, and 2.17 times less as compared to that observed in the control treatment under Cr stress (Cr-25). The Cr accumulation in soil was maximum (20.33 mg kg^–1^ soil) with the application of co-composted BC and it was 1.52 times more as compared to that observed in the control treatment without any amendment under Cr stress (Cr-25). Moreover, significant positive and negative correlations were observed among Cr concentration in plant parts and growth and physiological attributes of *Brassica* (Figure 6).

### Phytostabilization Efficiency and Health Risk Assessment

**FIGURE 7:**
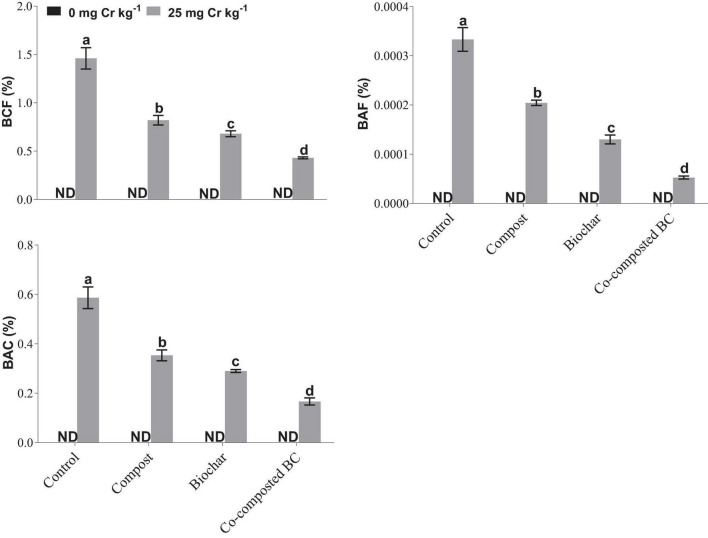
Impact of compost, biochar, and co-composted biochar on bioconcentration factor (BCF), bioaccumulation factor (BAF), and bioaccumulation concentration (BAC) of *Brassica napus*. The values are presented as mean ± standard error (*n* = 3). The values sharing the same letter(s) in bars are statistically non-significant with each other at *p* < 0.05.

**FIGURE 8:**
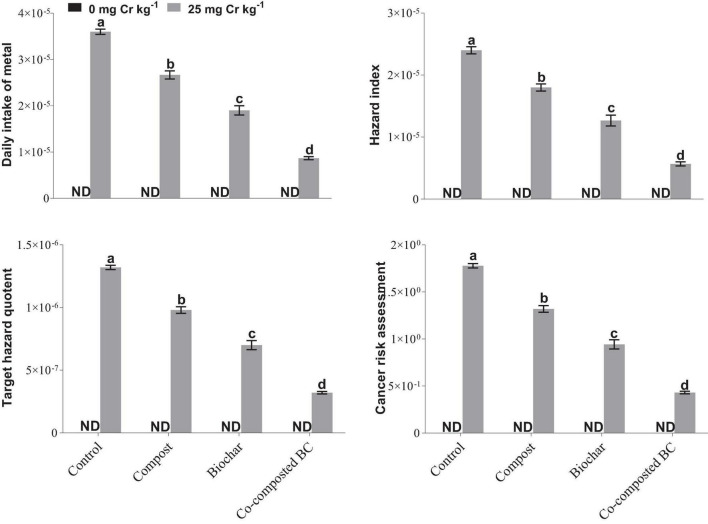
Impact of compost, biochar, and co-composted biochar on health risk assessment for the accumulation of Cr in *Brassica napus*. The values are presented as mean ± standard error (*n* = 3). The values sharing the same letter(s) in bars are statistically non-significant with each other at *p* < 0.05.

To estimate the phytostabilization potential of different amendments, bioconcentration factor (BCF), bioaccumulation factor (BAF), and bioaccumulation concentration (BAC) of *Brassica* were calculated (Figure 7). The application of compost, BC, and co-composted BC showed significant potential for the phytostabilization of Cr in the soil in comparison to the control treatment. The minimum values of BCF (0.43%), BAF (0.0000526%), and BAC (0.1667) were recorded with the application of co-composted BC as compared to the control under Cr stress (Cr-25). The health risk assessment was determined by calculating the daily intake of metal (DIM), hazard index (HI), cancer risk (CR), and total hazard quotient (THQ). The application of compost, BC, and co-composted BC significantly reduced the health risks associated with the accumulation of Cr in the edible portion of *Brassica* (Figure 8). With the application of co-composted BC, the minimum values of DIM (8.667 × 10^–6^), HI (5.667 × 10^–6^), CR (0.431), and THQ (3.2 × 10^–7^) were recorded, and these were 4.21, 4.24, 4.00, and 4.00 times less as compared to the control treatment without any amendment under Cr stress (Cr-25).

## Discussion

Results showed that the application of compost, BC, and co-composted BC significantly (*p* < 0.05) improved the growth attributes of *Brassica* in uncontaminated and contaminated soil (Cr-25). The soil under Cr stress (Cr-25) showed reduced shoot and root length, shoot fresh and dry weight, and root fresh and dry weight of *Brassica* (Figure 1). Many researchers have reported reduced morphological parameters under Cr stress (Adrees et al., 2015; Maqbool et al., 2018). Cr toxicity inhibits cell division and elongation in plant root cells (Adrees et al., 2015), thus reducing root surface area for enhanced water and nutrient uptake from the soil (Medda and Mondal, 2017; Ahmad et al., 2020) and ultimately growth and biomass production. Our findings are in line with the study by Jun et al. (2009) that Cr-contaminated soil reduced shoot length, root growth, and biomass production in cereals, vegetables, and forages.

Our results showed that plant growth significantly improved with the application of compost, BC, and co-composted BC. Novak et al. (2019) observed growth improvement when different combinations of BC and compost were used under Cd-contaminated soil. Abbas et al. (2020) observed that a combination of BC (3%) and AMS (5%) increased shoot length (93%), root length (222%), shoot dry weight (60%), and root dry weight (164%) of maize grown in Cr-contaminated soil. Arshad et al. (2017) reported that the pine wood chip BC produced at 450°C significantly improved maize root and shoot fresh weight, root length, and root surface area. Similarly, various studies have reported that BC application improved plant growth under a Cr stress environment (Agegnehu et al., 2017; Arshad et al., 2017). BC addition improves nutrient retention and water uptake and increases organic carbon (Rizwan et al., 2016; Cheng et al., 2020), thus enhancing soil fertility and boosting vegetative growth (Ali et al., 2018). In addition, the combined compost and BC influenced soil properties, such as pH, cation exchange capacity (CEC), soil organic matter, and nutrients availability, and ultimately increased soil fertility and plant growth (Agegnehu et al., 2017). In this study, an increase in plant growth parameters might be due to decreased Cr bioavailability with enhanced nutrient uptake under BC-amended Cr-contaminated soil. It may also be the BC -mediated conversion of Cr(VI) to Cr(III) in soil, which is relatively less mobile and less toxic (Rizwan et al., 2016).

Cr toxicity severely affects plant physiology and our results showed that the application of compost, BC, and co-composted BC significantly (*p* < 0.05) improved physiological attributes of *Brassica* in soil under Cr stress (Cr-25). Cr toxicity severely affects chlorophyll contents by disrupting chloroplast and discontinuing electron transport chain reaction, relative water contents, and electrolyte leakage in leaves. Many studies have shown that Cr toxicity damages photosynthetic machinery and produces a large amount of ROS in plants, which results in reduced growth (Ali et al., 2018; Bashir et al., 2020a).

Results of this study demonstrated that the application of compost, BC, and co-composted BC significantly reduced Cr uptake from soil under Cr contamination (Cr-25). Mustafa et al. (2020) observed that BC applied at the rate of 1 and 2% (w/w) reduced Cr uptake by 21 and 41%, respectively, under 500 μM of Cr stress. Similarly, Ali et al. (2018) observed that the application of BC efficiently removed Cr(VI) from wastewater. Cheng et al. (2020) noticed bamboo BC reduced Cu, Ni, Hg, and Cr uptake from water and soils. The reduction in Cr uptake might be due to the high surface area of BC that facilitates sorption, ion exchange, electrostatic interaction, complexation, precipitation, and co-precipitation (Rizwan et al., 2016). BC surface contains phenolic, hydroxyl, and carboxylic functional groups that facilitate metal binding (Mehmood et al., 2021; Murtaza et al., 2021a).

Under normal conditions, plants produce ROS in a controlled amount but under stress conditions; their production increases many times, which leads to oxidative stress, membrane permeability, DNA damage, and even cell death (Ahmad et al., 2010, 2019; Kohli et al., 2019). Plants have developed certain antioxidant enzymes to scavenge ROS and produce resistance against stress (Ditta and Khalid, 2016; Niamat et al., 2019). It was observed in many studies that the application of organic amendments enhanced resistance of plants against abiotic stresses (Seneviratne et al., 2017; Ali et al., 2018; Niamat et al., 2019). Our results showed that the application of co-composted BC indirectly influenced the antioxidant activity by reducing Cr bioavailability. In our findings, CAT activity was increased under Cr stress (Cr-25) in the control treatment. BC addition increased CAT activity (38.79%) in *Zea maize and Brassica rapa* (Ali et al., 2018; Bashir et al., 2020b). Similarly, Arshad et al. (2017) observed significant reductions in antioxidant enzyme activities under increasing levels of Cr stress. BC addition markedly improved antioxidant enzyme activities under Cr stress, which might be due to reduced Cr uptake (Seneviratne et al., 2017). Our results showed CAT, GSH, GR, GP, GPX, and GST activities in *Brassica* leaves increased under Cr stress (Cr-25), and the application of compost, BC, and co-composted BC reduced antioxidant activities. As discussed earlier, it was observed increased antioxidants activity in *B. napus* under Cd-spiked soil (Sabir et al., 2020).

This study results showed the co-composted BC significantly reduced Cr uptake in the *Brassica* plants. Novak et al. (2019) observed that the combined BC and compost reduced Cd uptake in plants. Seneviratne et al. (2017) also noted a significant reduction in cadmium (19.4%) and lead (22.0%) uptake when BC at the rate of 40 t ha^–1^ was applied in *Vigna radiata*. It was observed by many researchers that BC reduced HMs bioavailability by manipulating soil pH, precipitation, sorption, and changing HMs redox state (Ditta and Khalid, 2016; Rizwan et al., 2016; Cheng et al., 2020). BC contains a variety of oxygen-containing functional groups (such as carbonyl, carboxylic, hydroxyl, and phenol) that might be involved in complexation with HMs and cation exchange process with metals cations present on BC surface (Seneviratne et al., 2017; Niamat et al., 2019). With time, BC undergoes an oxidation process and new reactive sites are formed on BC surface that facilitates HMs immobilization (Bian et al., 2014) and ultimately results in reduced HMs uptake in plants. Such mechanisms of applied amendments might have resulted in reduced values of metal uptake in *Brassica* plants grown in this study and hence improved phytostabilization efficiency (Figure 7) and reduced health-associated risks (Figure 8). This was further evidenced by the negative correlations observed among Cr concentration in roots and shoots and growth and physiological parameters recorded for *Brassica* (Figure 6). The BCF and BAC values have further shown that the *Brassica* has the potential to extract Cr at higher concentrations and hence can be grown in metal-contaminated soils. Moreover, the values for DIMs and health risk index (HRI) were <1, which indicated that the consumption of *Brassica* was safe with no associated health risks.

## Materials and Methods

### Soil Sampling and Analysis

The contamination-free surface soil (0–15 cm) was collected from the research area of the Institute of Soil and Environmental Sciences, University of Agriculture Faisalabad. The soil was air-dried, ground, and sieved (2 mm) to remove stones and other undesired plant materials. Composite sampling was followed for soil homogenization. For pre-sowing analysis, the soil sample was taken from the composite sample.

Standard procedures were followed for basic soil analysis regarding physicochemical properties. Soil pH was determined by making a saturated soil paste of 250 g soil and pH was measured with a pH meter (Kent Eil 7015) (US Salinity Laboratory Staff, 1954). The electrical conductivity (EC) of soil was determined by taking soil extract from saturated soil paste, and extract EC was determined using EC meter (US Salinity Laboratory Staff, 1954). Soil texture was determined with the hydrometer method, and soil organic matter was determined by the potassium dichromate method (Moodie et al., 1959). Soil CEC was determined by the ammonium acetate method (Chapman, 1965), total soil nitrogen was determined through the Kjeldahl method (Jackson, 1962), soil available P by using NaHCO_3_ (Watanabe and Olsen, 1965), and soil K was determined through ammonium acetate (1N, pH = 7.0) method (US Salinity Laboratory Staff, 1954). Different physicochemical properties of soil are provided in Table 1.

**TABLE 1:** Physicochemical characteristics and nutritional composition of compost, biochar, co-composted biochar, and soil utilized in this study.

Physical/chemical properties	Soil	Compost	Biochar	Co-composted biochar
Textural class	Sandy clay loam	–	–	–
Sand (%)	56 ± 3.21	–	–	–
Silt (%)	24 ± 1.86	–	–	–
Clay (%)	20 ± 1.57	–	–	–
pH	7.88 ± 0.35	6.85 ± 0.03	7.964 ± 0.08	7.01 ± 0.09
Electrical conductivity (dS m^–1^)	1.06 ± 0.08	1.2 ± 0.01	2.75 ± 0.07	1.58 ± 0.08
Cation exchange capacity (cmol_*c*_ kg^–1^)	6.85 ± 1.62	73.01 ± 2.11	98.46 ± 2.86	92.35 ± 2.38
Moisture %	26 ± 1.26	10.57 ± 1.18	4.25 ± 0.19	7.32 ± 1.08
Organic matter (%)	0.72 ± 0.18	–	–	–
Carbon (g kg^–1^)	–	199.2 ± 3.68	633.51 ± 5.67	398.92 ± 4.33
Nitrogen (g kg^–1^)	–	21.8 ± 1.26	11.25 ± 0.86	16.42 ± 0.67
Total phosphorus (g kg^–1^)	–	0.42 ± 0.05	1.43 ± 0.54	1.18 ± 0.67
Total potassium (g kg^–1^)	–	1.67 ± 0.18	9.29 ± 0.22	7.32 ± 0.63
Zinc (mg kg^–1^)	–	47.0 ± 3.70	45.61 ± 2.91	46.27 ± 2.87
Iron (mg kg^–1^)	–	70.8 ± 4.09	85.31 ± 4.76	82.29 ± 3.67

*The values are presented as mean ± SE (n = 3).*

For bioavailable Cr concentration, the soil sample (10 g) was extracted with AB-DTPA solution (20 ml) and shaking at 180 rpm on a reciprocal shaker for 15 min (Soltanpour and Schwab, 1977). Concentrated HNO_3_ (100 ml) was added to the extract and analyzed for bioavailable Cr using atomic absorption spectrophotometer (PerkinElmer Model 700, United States). For total Cr concentration in soil, the method proposed by Soltanpour and Schwab (1977) was adopted. Briefly, soil samples (0.5 g) were digested with 15 ml of three acids mixture (HNO_3_, H_2_SO_4_, and HClO_4_ in a ratio of 5:1:1) and heated at 160°C in digestion chamber until a colorless solution appeared. After digestion, the samples were cooled, filtered, and diluted to a 50-ml solution using a volumetric flask with deionized water. An atomic absorption spectrophotometer (PerkinElmer Model 700) was used for the determination of total Cr concentration in the samples.

### Compost, Biochar, and Co-composted Biochar Production and Characterization

For compost preparation, a locally fabricated composting unit was used involving animal manure as an easily available raw material (Ditta et al., 2015, 2018a,b). The animal manure was collected from Animal Husbandry Farm, the University of Agriculture, Faisalabad, and sun-dried to remove excess moisture and unwanted plant debris, stone, and plastic material. During composting, 50% moisture at 65°C was maintained with 50-rpm speed and this process was completed after 40 days. Co-composted BC was prepared by mixing 30% (w/w) wheat straw BC with composted material during the last week of composting. For wheat straw BC preparation, straw feedstock was collected from the field area of the Department of Agronomy, University of Agriculture Faisalabad, washed with tap water to remove dust, sun-dried, and crushed. Sanchez et al. (2009) described a process that was followed for BC production in which feedstock was filled in a Pyrex flask having 2 L capacity. BC was prepared in a muffle furnace (Gallonhop, England) at 400°C under an oxygen-limited environment, with 20 min of retention time at heating at the rate of 10°C min^–1^ increase in temperature. Gases and water vapor produced during pyrolysis were removed by using a silicon-made bent glass rod attached to the flask outlet. After pyrolysis, the furnace remained closed until 20°C, and the prepared BC was stored in plastic bags for further analysis.

For pH and EC, each compost, BC, and co-composted BC was separately mixed with deionized water in 1:20 ratio (w/v), shaken for 1 h, and pH and EC were measured using pH meter (PHS-38W) and EC meter (4510 conductivity meter). Nutrient analysis was carried out through wet digestion of each sample using H_2_SO_4_ and H_2_O_2_ (Wolf, 1982). In 1 g of each sample, H_2_SO_4_ (15 ml) was added and placed in the sample overnight. On the next day, the digestion mixture was heated on a hotplate until the boiling started. In the boiling digestion mixture, H_2_O_2_ (10 ml) was added repeatedly until a colorless solution appeared. The volume of the digested sample was made 50 ml in a volumetric flask using deionized water and preserved until the analysis was performed. Total nitrogen was determined through the Kjeldahl method (US Salinity Laboratory Staff, 1954), P was measured by NaHCO_3_ method using a spectrophotometer (UV-1201, Shimadzu, Tokyo, Japan), and K was determined using a flame photometer (PFP7, Jenway, Essex, United Kingdom). Different physicochemical properties of compost, BC, and co-composted BC are given in Table 1.

### Pot Experiment

After the preparation of different organic amendments, a pot experiment was conducted in rain-protected wirehouse of Institute of Soil and Environmental Sciences (31.439052^°^ N, 73.069335^°^ E), University of Agriculture Faisalabad, Pakistan. In each pot, 10 kg soil was used and recommended rate of NPK (90:60:75 kg ha^–1^) using urea, di-ammonium phosphate, and sulfate of potash fertilizer was mixed. Compost, BC, and co-composted BC were applied at the rate of 2% (w/w). For Cr contamination, the soil was spiked with potassium dichromate (K_2_Cr_2_O_7_) solution at two levels (0 and 25 ppm) and applied to plants at a three-leaf stage after complete germination. In each pot, six seeds of *B. napus* (cv. AARI canola) were sown, and each treatment was applied in triplicate. The pots were placed in the wirehouse under natural conditions as day/night temperature of 25°C/15°C and relative humidity of 50%/70%, respectively. Recommended agronomic practices were followed during the course of the study. The harvesting was done after 60 days of plant growth at maturity. Data regarding growth, physiological, and biochemical parameters were measured as follows.

### Growth and Physiological Parameters

After harvesting the plants, data regarding various growth and yield parameters were taken following standard procedures. For shoot and root length, measuring tape was used. For fresh biomass, the root and shoot samples were weighed on a digital balance. For dry biomass, the samples were put in a drying oven at 65°C for 48 h or until a constant weight was obtained. The shoot samples with grain pods were dried in sun. The threshing of shoot samples was done to separate grains, and the grain yield was measured by weighing the grains on a digital balance. Plant physiological attributes such as photosynthetic rate, transpiration rate, and stomatal conductance were measured by using an infrared gas analyzer (IRGA; Analytical Development Company, Hoddesdon, England) at morning time (Ben-Asher et al., 2006). Chlorophyll contents were measured in the morning time with a portable chlorophyll meter (SPAD-502, Konica-Minolta, Japan) following the method suggested by Arnon (1949), while relative water contents were measured using the method suggested by Barrs and Weatherley (1962).

To assess membrane permeability against Cr stress, electrolyte leakage was determined following the method suggested by Lutts et al. (1996). Briefly, the youngest fully expanded leaf sample (1 cm) from each replicate was taken into a vial (10 ml) and washed three times with distilled water to remove any dust material. Each vial with leaf sample was placed in the shaker (100 rpm) at room temperature (25°C) for 24 h. On the following day, the EC of the leaf sample in the vial was measured using an EC meter and denoted as EC_1_. Then, each leaf sample in the vial was autoclaved at 121°C for 20 min. After cooling the samples, again EC was measured using an EC meter and denoted as EC_2_. The electrolyte leakage was determined by using the following formula:


$$\mathrm{Electrolyte}\mathrm{leakage}\left(\%\right)=\frac{E\,C_{1}}{E\,C_{2}}\times 100$$

### Antioxidant Enzyme Activity

For different antioxidant enzyme activities, fully expanded, uppermost fresh leaf samples after 40 days of germination were used. For this purpose, 0.5 g leaves were homogenized in 3 ml Tris buffer (50 mM) having pH 7.8, centrifuged at 12,000 rpm for 20 min, and the total soluble enzyme activities were calculated by using a spectrophotometer. Leaf CAT activity was measured following the method suggested by Aebi (1983), while ascorbate peroxidase activity was measured by using a spectrophotometer at 290 nm (Nakano and Asada, 1981). The method suggested by Fryer et al. (1998) was used for the determination of glutathione reductase and that by Rahman et al. (2006) for the measurement of reduced glutathione activity, in which DTNB (5,5′-dithiobis-2-nitrobenzoic acid) reduced to TNB (2-nitro-5-thiobenzoic acid) and changed the absorbance. For glutathione peroxidase activity, the method suggested by Rahman et al. (2006) was followed. Similarly, the methodology suggested by Habig et al. (1974) was followed for glutathione-*S*-transferase activity in leaf samples.

For the determination of TSC, the method suggested by Yem and Willis (1954) was used. Briefly, 0.5 g of ground leaf samples were extracted in ethanol solution (80%). Then, 10 ml of extract solution was taken in a 25-ml test tube along with 6 ml anthrone reagent and heated in the water bath for 10 min. Anthrone reagent was freshly prepared by taking 150 mg of anthrone in 72% pure H_2_SO_4_. Heated samples were cooled for 10 min, and the absorbance was measured at 625 nm using a spectrophotometer (UV-1201, Shimadzu, Tokyo, Japan).

### Evaluation of Phytoimmobilization Efficiency

For BAC and BCF, the following equations were used to assess the phytoimmobilization/phytostabilization potential of *B. napus* as proposed by Amin et al. (1954) and Zhuang et al. (2007).


$$\text{BAC}=\frac{\text{Ni}\,\left[\text{shoot}\right]}{\text{Ni}\,\left[\text{soil}\right]}$$

where Ni [shoot] and Ni [soil] are the concentrations of Ni in the shoots and the soil, respectively.


$$B\,C\,F=\frac{M\,\left(h\,\mathrm{arvested}\,\mathrm{tissue}\right)}{M\,\left(\mathrm{soil}\,\mathrm{water}\right)}$$

where *M* (harvested tissue) is the metal concentration in the harvested plant parts (root, shoot, and grains), and *M* (soil water) is the total amount of applied metal for the corresponding treatment.

### Intake of Heavy Metals From Vegetables and Health Risk Assessment

The DIM index was calculated by using the following equation (Latif et al., 2018):


$$D\,I\,M=\frac{M\times I\times K}{W}$$

where *M* is the concentration of HMs in plants (mg kg^–1^), *K* is the conversion factor, *I* is the daily intake of vegetables, and *W* is the average body weight (BW). The average adult BW was considered 60 kg, while the average daily vegetable intake for adults was considered 0.345 kg^–1^ person^–1^ (Latif et al., 2018).

The HRI for Ni caused by the consumption of contaminated vegetables was calculated using the following equation (Jan et al., 2010):


$$H\,R\,I=\frac{D\,I\,M}{R\,f\,d}$$

where *DIM* is the daily intake of metals and *Rfd* is the reference oral dose. According to US-EPA IRIS, the *Rfd* value for Ni is 0.02 mg kg^–1^ BW day^–1^.

### Statistical Analysis

Data collected were evaluated using analysis of variance (ANOVA), and treatment means were compared by using Tukey’s HSD test at 5% probability level using Statistix 8.1 (Analytical Software, Tallahassee, FL, United States) (Little and Hills, 1978). The figures were plotted using GraphPad Prism computer-based software (GraphPad Software, San Diego, CA, United States). The correlation matrix was computed using R software (https://www.R-project.org/).

## Conclusion

The results showed that Cr stress (Cr-25) significantly reduced growth, physiological and biochemical parameters of *Brassica* under the control treatment without any amendment. The application of compost, BC, and co-composted BC significantly alleviated Cr stress and enhanced various growth, physiological, and biochemical parameters. With the application of co-composted BC, the maximum increase in growth, physiological, and biochemical parameters of *Brassica* was recorded in comparison to the control treatment without any amendment under Cr stress (Cr-25). Similarly, the application of co-composted BC maximized the phytostabilization efficiency as revealed through the minimum values of BCF, BAF, and BAC of *Brassica* as compared to the control treatment under Cr stress (Cr-25). Moreover, the same treatment significantly reduced the health risks associated with the consumption of *Brassica* under Cr stress (Cr-25) as clear from the lowest values of DIM, HI, CR, and THQ. In conclusion, the application of co-composted BC could serve as an effective amendment in the alleviation of Cr stress in *Brassica* via phytostabilization and reduction in associated health risks.

## Data Availability Statement

The original contributions presented in the study are included in the article/supplementary material, further inquiries can be directed to the corresponding authors.

## Author Contributions

AM, WX, and MN: conceptualization. BT: methodology. BT, AD, and MB: software. AM, MN, and MZN: validation. MB and JK: formal analysis. ZS and QS: resources. BT and QS: data curation. MN: writing—original draft preparation. AM, JK, and MR: writing—review and editing. AM and WX: supervision. MN and AD: project administration. WX and MB: funding acquisition. All authors have read and agreed to the published version of the manuscript.

## Conflict of Interest

The authors declare that the research was conducted in the absence of any commercial or financial relationships that could be construed as a potential conflict of interest.

## Publisher’s Note

All claims expressed in this article are solely those of the authors and do not necessarily represent those of their affiliated organizations, or those of the publisher, the editors and the reviewers. Any product that may be evaluated in this article, or claim that may be made by its manufacturer, is not guaranteed or endorsed by the publisher.
